# Development of a method for qualitative data integration to advance implementation science within research consortia

**DOI:** 10.1186/s43058-025-00701-4

**Published:** 2025-02-25

**Authors:** Lisa DiMartino, Allison J. Carroll, Jennifer L. Ridgeway, Anna Revette, Joan M. Griffin, Bryan J. Weiner, Sandra A. Mitchell, Wynne E. Norton, Christine Cronin, Andrea L. Cheville, Ann Marie Flores, Justin D. Smith, the IMPACT Consortium, Lisa DiMartino, Jennifer L. Ridgeway, Joan M. Griffin, Bryan J. Weiner, Sandra A. Mitchell, Wynne E. Norton, Christine Cronin, Andrea L. Cheville, Ann Marie Flores, Justin D. Smith, David Cella, Michael J. Hassett, Raymond U. Osarogiagbon, Deborah Schrag, Sandra L. Wong, Barbara L. Kroner, Ashley Wilder Smith, Sofia Garcia, Roxanne Jensen, Kathryn Ruddy, Betina Yanez, Jessica J. Bian, Don S. Dizon, Hannah W. Hazard-Jenkins, Mary-Anne Ardini, Paige Ahrens, Jessica Austin, Fiona Barrett, Michael Bass, Megan Begnoche, September Cahue, Kimberly Caron, Linda Chlan, Ava Coughlin, Samira Dias, Nicolas Faris, Martha Garcia, Karla Hemming, Jeph Herrin, Christine Hodgdon, Sheetal Kircher, Kurt Kroenke, Veronica Lam, Nicola Lancki, Quan H. Mai, Jennifer Mallow, Nadine J. McCleary, Mary O’Connor, Deirdre Pachman, Loretta Pearson, Frank Penedo, Jewel Podratz, Jennifer Popovic, Liliana Preiss, Parvez Rahman, Sarah Redmond, James Reich, Joshua Richardson, Kimberly Richardson, Lila Rutten, Karen Schaepe, Denise Scholtens, Tiana Poirier-Shelton, Philip Silberman, Jaclyn Simpson, Laura Tasker, Nathan Tesch, Cindy Tofthagen, Angela Tramontano, Benjamin D. Tyndall, Hajime Uno, Firas Wehbe

**Affiliations:** 1 Peter O’Donnell Jr. School of Public Health, University of Texas Southwestern Medical Center, Dallas, TX USA; 2 RTI International, Research Triangle Park, NC USA; 3 Department of Psychiatry and Behavioral Sciences and Medical Sciences, Center for Dissemination and Implementation Science, Northwestern University Feinberg School of Medicine, Chicago, IL USA; 4 Robert D. and Patricia E. Kern Center for the Science of Health Care Delivery and Division of Health Care Delivery Research, Mayo Clinic, Rochester, MN USA; 5 Division of Population Sciences, Dana Farber Cancer Institute, Boston, MA USA; 6 Departments of Global Health and Health Systems and Population Health, University of Washington, Seattle, WA USA; 7 Division of Cancer Control and Population Sciences, National Cancer Institute, Bethesda, MD USA; 8 Department of Physical Medicine and Rehabilitation, Mayo Clinic, Rochester, MN USA; 9 Departments of Physical Therapy and Human Movement Sciences and Medical Social Sciences, Northwestern University Feinberg School of Medicine, Chicago, IL USA; 10 Robert H. Lurie Comprehensive Cancer Center, Northwestern University, Chicago, IL USA; 11 Department of Population Health Sciences, Spencer Fox Eccles School of Medicine, University of Utah, Salt Lake City, UT USA; 12 Departments of Psychiatry and Behavioral Science and Medical Social Sciences, Northwestern University Feinberg School of Medicine, Chicago, IL USA

**Keywords:** Cancer, Implementation science, Qualitative methods, Consolidated Framework for Implementation Research, Data integration

## Abstract

**Background:**

Methods of integrating qualitative data across diverse studies and within multi-site research consortia are less developed than those for integrating quantitative data. The development ofsuchmethods is essential to support the data exchange needed for cross-study qualitative inquiry and given the increasing emphasis on data sharing and open science. We describe methods for qualitative data integration within the National Cancer Institute’s Improving the Management of symPtoms During And following Cancer Treatment (IMPACT) Consortium funded by the Cancer Moonshot^SM^. Data collection and analysis were guided by the Consolidated Framework for Implementation Research (CFIR). Our case study highlights potential solutions for unique challenges faced when integrating qualitative data across multiple settings in a research consortium.

**Methods:**

The IMPACT consortium is comprised of three research centers (RCs) each conducting pragmatic trials examining the effectiveness of routine symptom management on patient-centered outcomes. After reaching consensus on use of CFIR as the common implementation determinant framework, RCs developed a semi-structured interview guide and tailored it to features of their healthcare setting and symptom management interventions. RCs conducted interviews/focus groups with healthcare system partners to examine contextual factors impacting implementation. RCs exchanged 1–2 transcripts (n = 5 total) for purposes of pilot testing the methodology.

**Results:**

Given the heterogeneity of study settings and contexts, it was challenging to simultaneously assign codes at both domain and construct levels and the process was resource intensive. Recommendations include employing a common framework for data collection and analyses from the outset, coding at domain level first and then incorporating construct codes, and centralizing processes via a coordinating center (or similar entity) and combining coded transcripts using qualitative software. We also generated an iteratively refined codebook that employed the CFIR schema and incorporated CFIR 2.0 to provide detailed guidance for coders conducting cross-study qualitative inquiry.

**Conclusions:**

Limited guidance exists on how to support qualitative data integration, data exchange, and sharing across multiple studies. This paper describes a systematic method for employing an implementation determinant framework-guided approach to foster data integration. This methodology can be adopted by other research consortia to support qualitative data integration, cross-site qualitative inquiry, and generate improved understanding of evidence-based intervention implementation.

**Supplementary Information:**

The online version contains supplementary material available at 10.1186/s43058-025-00701-4.

Contributions to the literature

- Few standard practices exist for integrating qualitative data across different studies to conduct cross-study qualitative inquiry. Developing data integration procedures is critical to generating insights about practice improvement across settings and achieving goals of open science.
- This report describes a method for integrating qualitative data using the Consolidated Framework for Implementation Research illustrated within the context of NCI’s Improving the Management of symPtoms During And following Cancer Treatment (IMPACT) Consortium.
- The method describedcan be applied within research consortia and across multi-site studies to support qualitative data integration, thereby producing valuable insights to improve implementation of evidence-based interventions.

## Background

While methods for standardizing quantitative data collection and analysis across research studies through the use of common data elements, i.e., common measures, data collection procedures, and coding/variable construction, are well established [1, 2], there are few standard practices for how to support integration of qualitative data across studies [3]. Although other definitions exist [4, 5], in this paper “data integration” refers to the process of combining qualitative data to compare experiences across multiple studies and different care delivery contexts. Unique challenges to qualitative data integration exist, including non-standardized and varied data collection methods, large volumes of textual data, setting-specific protocols, and context-dependent analytic procedures that may require extensive interpretation. Yet, developing methods and procedures to exchange and integrate qualitative data is critical to ensuring data are collected and coded in a rigorous manner and suitable for use in conducting qualitative inquiry across studies and populations to generate valuable insights about the process of implementing evidence-based interventions (EBIs) into practice [6]. The importance of developing these methods is also underscored by the movement towards the principles and practice of open science [6, 7]. For example, the National Institutes of Health (NIH) recently revised data sharing policy which now includes qualitative data [8]. Public data sharing, and the subsequent secondary analysis of qualitative data, is not normative among researchers in the social and behavioral sciences, and even less so in implementation science [7, 9, 10]. To this end, the National Cancer Institute (NCI) has developed a framework for equitable implementation data sharing as this issue takes on greater emphasis in the field [11].

In recent years, the NIH has funded several research consortia, where the inclusion of qualitative data on implementation context contributes to the understanding, uptake, and sustainability of EBIs [12–14]. Research consortia offer unique opportunities to build an evidence base for implementation of EBIs in diverse settings as well as to develop common methods, data collection instruments, and analytic approaches that facilitate data exchange and support generation of robust evidence. Our research consortium is the NCI’s Improving the Management of symPtoms During And following Cancer Treatment (IMPACT) Consortium funded by the Cancer Moonshot^SM^ to conduct trials that use implementation science methods to accelerate adoption of electronic health record (EHR)-integrated patient reported outcome (ePRO) systems and evidence-based symptom management [15]. In this paper, we provide a case example and outline the steps involved in developing a method for qualitative cross-study coding to support consortium-wide data integration. Specifically, this article describes use of the Consolidated Framework for Implementation Research (CFIR) for data collection and coding and offers solutions to address unique challenges that arise when integrating qualitative data across diverse studies within the structure of a research consortium.

## Methods

IMPACT includes three Research Centers (RCs) and a coordinating center. Details about the design and methods of these three large pragmatic trials are provided elsewhere [15–20]. The Implementation Science Workgroup (ISWG) was charged with integrating qualitative data across RCs. As the RCs had independently designed their studies, each had initially proposed a distinct implementation determinant framework to qualitatively examine contextual factors (e.g., barriers and facilitators) impacting implementation. The frameworks originally were: Normalization Process Theory [21], CFIR [22], and the Exploration, Preparation, Implementation, and Sustainment (EPIS) framework [23]. The RCs employed a multi-step collaborative decision-making process that involved reviewing the originally proposed frameworks and reaching consensus on a shared framework to enable comparisons across the studies [24]. The RCs decided to use the CFIR to guide data integration efforts because of its comprehensiveness, and because many of the domains included in CFIR subsume domains found in the other frameworks.

**Fig. 1:**
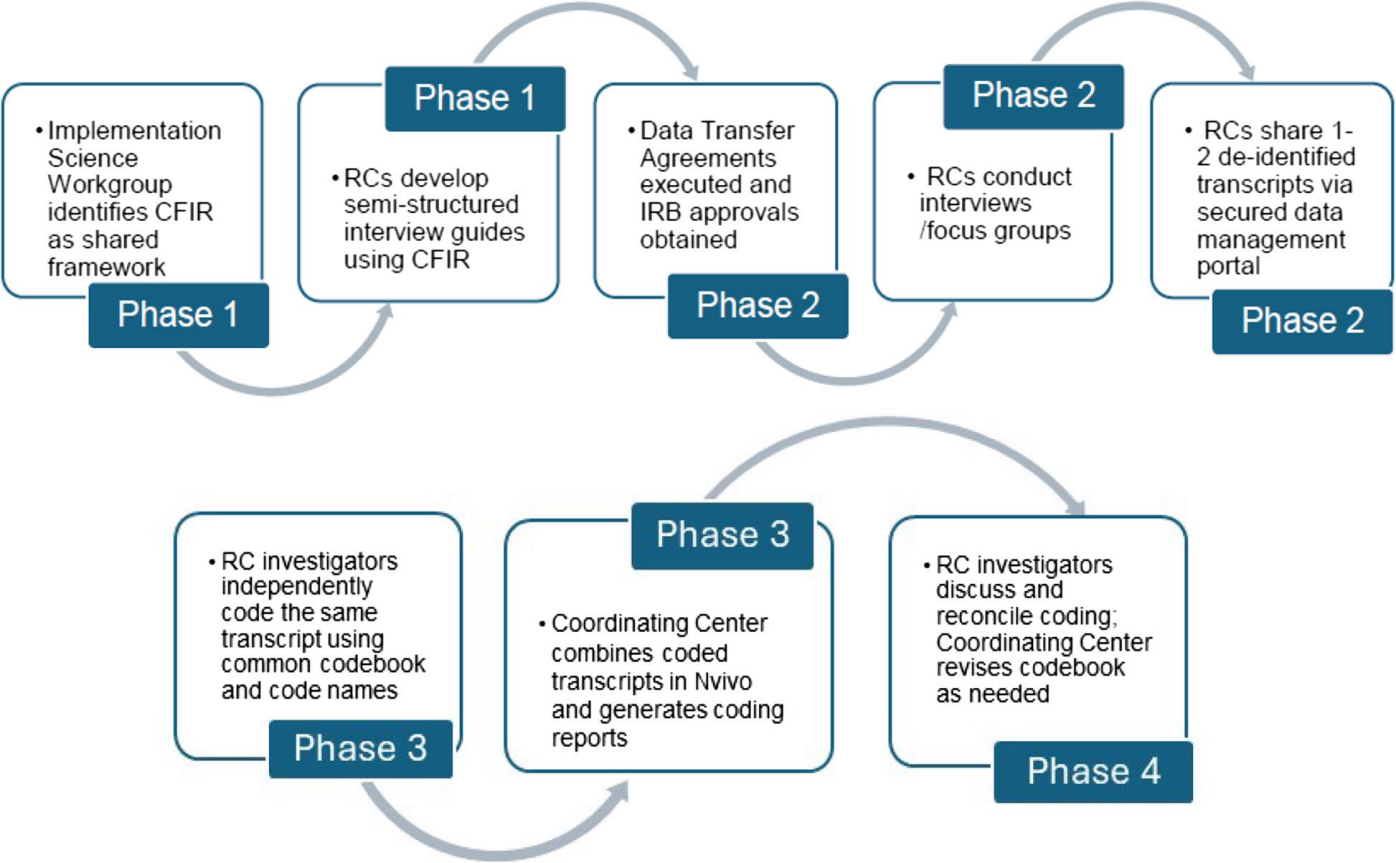
Implementation Science Workgroup steps and procedures for integrating qualitative data across the IMPACT consortium. RC, Research Center; IRB, Institutional Review Board

Each RC developed a semi-structured interview guide tailored to the features of their specific healthcare settings, the interventions being tested, and scaffolded by CFIR domains and constructs. This approach ensured a common structure for data collection and attention to comparable implementation determinants across studies. Of note, one RC used the updated CFIR 2.0 because their data collection began after CFIR 2.0 was published [25]. CFIR 2.0 is based on the original framework and contains many of the same constructs that can be mapped back to the original CFIR. All three of the RC’s interview guides included questions from each CFIR Domain (Innovation, Outer Setting, Inner Setting, Individuals, and Process). Figure 1 provides a summary of the ISWG data integration steps and procedures. Further details of our methodology are provided in Additional File 1.

## Results

These efforts to develop and pilot test a methodology to integrate qualitative data across three RCs revealed several considerations that can inform similar efforts:

- During study design, the team should identify a common implementation determinant framework for data collection and analyses prior to constructing the interview guide, conducting interviews, or analyzing the data.
- The team should pilot test an initial transcript to ensure investigators have a common understanding of what data elements to code, how each code is defined, and fluency in applying the definitions to transcript excerpts. Although investigators had experience using CFIR, we used the first transcript as a “trial run” where coding was done deductively at the domain level first (e.g., “*Intervention Characteristics*”) and then constructs (e.g., “*Innovation Adaptability*”) were incorporated. Other groups have used a similar approach to prospectively maintain consistency in coding and support coders in efficiently reaching agreement in the coding process [26].
- Future research consortia should employ a coordinating center (or similar entity), or support an RC to assume this role, to help establish centralized processes including codebook development and maintenance, combining coded transcripts using qualitative software, and to facilitate collaboration across RCs in a consortium as they are performing coding activities and resolving discrepancies.
- If the interventions and/or implementation strategies being tested are highly disparate across studies, qualitative data integration across studies may be more challenging to achieve and would also be unlikely to generate meaningful information to guide future implementation efforts. Similarly, if the types of participants recruited for qualitative data collection are likely to have different experiences of the implementation determinants (i.e., patient perspectives versus health care system stakeholders) data integration may not be advisable.

In addition, our methodology generated a codebook designed to be disseminated to the RCs to promote a consistent method for coding shared qualitative data and addressing research questions about implementation of ePROs for cancer care (see Additional File 2). The codebook incorporates new constructs from CFIR 2.0 to allow for continuity of our data integration and ensure relevancy of our approach. Although all the coders were experienced qualitative researchers, challenges arose along the way due in part to different levels of experience using CFIR. This resulted in initial coding disagreements across many CFIR domains and constructs that were resolved through coding refinement and calibration. For example, during the coding process there was confusion across the studies about whether a transcript reference pertained to a component of the intervention itself (i.e., “*Intervention Characteristics*” domain), or to the implementation of the intervention (i.e., “*Process*” domain). To address this issue, each RC developed a table that clearly distinguished the intervention components (e.g., components related to the electronic collection of symptom data) and implementation strategies (e.g., clinical alerts to providers for severe symptoms), which was included in the codebook (see Additional File 2, pgs. 20–21).

Another challenge was navigating the “*Roles*” subdomain which was added to the “*Individuals*” domain as part of CFIR 2.0. Coders found it difficult to assign a CFIR-defined role for some clinicians (e.g., physicians, nurses, social workers), particularly because their roles (e.g. innovation deliverers, innovation recipients, implementation team members) differed across the studies, and in some cases one individual functioned in multiple roles (e.g. member of the care delivery team and an implementation team member). To address this issue, each RC specified which position(s) corresponded to each of the CFIR “*Roles*” and provided specific examples. Additional file 3 includes definitions and inclusion criteria for constructs within the *“Roles”* subdomain along with details of coding discrepancies that arose, and our approach to achieving a consensus around a shared interpretation and assignment of a code to a specific excerpt.

## Discussion

This Short Report illustrates with a case example the development of a method to integrate qualitative data across multiple centers within a research consortium. We used a common implementation determinant framework, CFIR, to shape our data collection and integration efforts. In marked contrast to quantitative data, limited guidance exists in the literature about methods to integrate qualitative data across individual research studies, including studies conducted as part of a research consortium. However, methods development to support qualitative data exchange and integration has become increasingly important given an increased shift among funders towards data sharing, desire for more open science, and the potential for qualitative data from multiple sites to yield valuable insights to inform EBI implementation [9]. Use of common interview questions facilitates subsequent cross-study analyses, but it is not always feasible to achieve that level of standardization within a consortium. Moreover, imposing that level of uniformity across diverse study contexts could detract from the overarching goal of including qualitative data. Qualitative methods are typically included in implementation studies to provide information about barriers and facilitators to EBI adoption, and to understand strategies and processes that are needed to support implementation of the EBI to achieve sustained adoption [3]. Importantly, our goal was not to develop a method for harmonizing the data elements, as it was not possible or desirable given the different sampling strategies and data collection processes across the studies. Thus, we provide here one method for integrating qualitative data across multiple studies using a common coding schema derived from CFIR.

We found our process was feasible yet challenging in several ways. Given the heterogeneity of qualitative research methods used (individual interviews versus focus groups, different participant inclusion criteria, and different semi-structured interview guides) there were some difficulties selecting the most appropriate codes at domain and construct levels. The introduction of CFIR 2.0 midstream amplified these complexities. However, we determined CFIR 2.0 would be useful for our data integration efforts because of the emphasis on specific roles and characteristics of individuals involved in the study and addition of relevant new subconstructs. In addition, investigators often noted challenges in independently coding transcripts before meetings because they lacked information about another RC’s EBI and/or implementation context, especially when participants referred to specific terms or roles only used in that setting. Meetings to review transcript coding served as opportunities for reflexive discussion between “insiders” and “outsiders” on each RC study. Investigators who were not involved in the work being described in the transcript asked probing questions that generated refection on what was happening in the data and the context, how CFIR codes were being applied, and what assumptions or perspectives shaped interpretation. This discussion provided additional details or examples for the codebook, and in some cases, investigators changed their original coding based on the discussion. Inclusion in the codebook examples of quotes from each transcript and describing reasons to select a specific code versus a related code also helped to address this challenge and improved coder agreement. Further, developing the methodology for data integration was time-consuming and resource intensive, and as such it may not be feasible for studies operating with limited time and funding. However, investment in this process was intended to help the RCs to come to agreement on coding schema, thereby facilitating more efficient and reliable coding efforts for cross-consortium analyses. Lastly, the interventions being evaluated in the IMPACT consortium were somewhat distinct in their approach, which introduced challenges in coding as described above. At the same time, while the specifics of the intervention form varied across RCs, all had the shared function of implementing ePROs surveillance in ambulatory oncology care. Consequently, the RCs had many common components, thereby aiding our data integration efforts.

Despite these challenges, our work has several important features that enhance rigor and reproducibility. First, implementation science offers many frameworks from which to assess implementation determinants. As described in previous research consortia, identification of a common framework is a critical first step in the process of establishing common measures [27, 28]. Our study adds to the literature by illustrating that use of a common framework, such as CFIR, prior to the commencement of the qualitative work can enhance standardization of measurement and data collection, coding, and support data exchange and integration in research consortia.

A second strength was our approach to code at a higher level first and then incorporate subconstructs into subsequent rounds of coding as the team became more familiar with construct definitions and the codebook was iteratively refined. This helped to ensure interpretive consistency of CFIR constructs and to establish an initial familiarity with the codes. In our case, CFIR provided the domains and subconstructs, but a similar approach could be used with other multi-layered implementation determinant frameworks, such as the Exploration, Preparation, Implementation, and Sustainment (EPIS) model [23], Practical Robust Implementation and Sustainability Model (PRISM) [29], and integrated Promoting Action on Research Implementation in Health Services (i-PARIHS) framework [30]. Our approach may also be applied to CFIR combined with Normalization Process Theory (NPT) for exploring more granular implementation processes [31].

As is the case with any qualitative study with multiple coders, it is important for the research team to build consensus on codebook definitions. To that end, an additional strength of our methodology was our development of pragmatic tools (e.g., tables) documenting study-specific features that may differ across the studies we were trying to integrate. For example, consistent with updated CFIR guidance [25], we developed a table that distinguished the *innovation* being implemented from the *implementation strategies* to ensure consistency in coding (see Additional File 1, pgs. 20–21). A similar process was undertaken to ensure consistency in coding for the “*Roles*” subdomain (see Additional File 3). Other studies may similarly need to develop a set of decision rules or guidelines as part of their coding schema to support agreement among coders and enhance reproducibility. A key next step will be to use the codebook developed in the IMPACT consortium to answer high priority research questions related to implementation of ePRO systems in cancer care.

There are some limitations that should be noted. First, one investigator from the coordinating center manually entered coding into Nvivo to allow for coding comparison. This was done due to lack of compatibility of different versions of Nvivo across the RCs. From a data management perspective, this was possible for our study given the small sample of transcripts but may not be feasible if there are many transcripts to be coded. Future consortia should plan for ways to optimize their workflows and utilize interoperable software solutions. Second, it is possible some CFIR constructs may not be represented in this sample of transcripts. Thus, some coding decisions that may have arisen with a larger sample of transcripts may not be fully addressed within the codebook. At the same time, we purposively sampled transcripts to ensure inclusion of transcripts from each RC, and selected transcripts with detailed textual data that would allow for broad application of CFIR constructs. Further, the objective of this work was not to achieve data saturation, but rather to develop a generalizable methodology that could be used by our consortium and other consortia for cross-study qualitative inquiry.

## Conclusions

There are many potential benefits to integrating qualitative data, including the ability to synthesize findings across studies to generate valuable insights to improve implementation of EBIs. However, historically, qualitative data have not been shared and cross-study analyses are infrequently conducted. We present a systematic approach for qualitative cross-study coding and data integration informed by CFIR. It is anticipated this approach can be applied across multi-site studies and within the structure of research consortia to support exchange and integration of qualitative data and public data sharing, generating new insights that achieve sustained adoption of EBIs in the delivery of healthcare to diverse individuals, settings, and communities.

## Supplementary Information

Supplementary Material 1.

Supplementary Material 2.

Supplementary Material 3.

## Data Availability

The de-identified interview transcripts that support the findings of this article are available from the corresponding author upon reasonable request; restrictions and some redactions may be applied to preserve the rights of participant confidentiality. All other materials applicable to this study are included in the published article and its supplementary information files.

## Abbreviations

CFIR: Consolidated Framework for Implementation Research
EPIS: Exploration, Preparation, Implementation, and Sustainment Framework
EBI: Evidence Based Intervention
IMPACT: Improving the Management of sympToms during And following Cancer Treatment
NCI: National Cancer Institute
NIH: National Institutes of Health
NPT: Normalization Process Theory
ePRO: Electronic Patient Reported Outcome
PRISM: Practical Robust Implementation and Sustainability Model
i-PARIHS: integrated Promoting Action on Research Implementation in Health Services
RC: Research Center
