# Supplementary material for: Development of a method for qualitative data integration to advance implementation science within research consortia
Source: Implement Sci Commun. 2025 Feb 25;6:21. doi: 10.1186/s43058-025-00701-4 (PMC11853699; doi:10.1186/s43058-025-00701-4)
Supplement: Supplementary file 1 — Supplementary Material 1. [file 43058_2025_701_MOESM1_ESM.docx]

**Additional File 1.**

**Methodology Details**

The Research Centers (RCs) of the IMPACT consortium are: (1) Northwestern University (NU) IMPACT (ClinicalTrials.gov ID NCT03988543); (2) Enhanced EHR-Facilitated Cancer Symptom Control (E2C2; ClinicalTrials.gov ID NCT03892967), and (3) Symptom Management IMplementation of Patient Reported Outcomes in Oncology (SIMPRO; ClinicalTrials.gov ID NCT03850912).

**Interview Guide Development.** All three interview guides included questions from each CFIR domain. For example, for the “*Innovation Domain*”, participants were asked about what other care team members generally thought about integrating ePROs into cancer care (SIMPRO), what evidence they were aware of that showed whether or not ePROs would work in their setting (NU IMPACT), and what they thought about the E2C2 intervention when they first heard about it. Each interview guide was reviewed by members of their respective RC team. Two investigators from the coordinating center (LD and BW) with expertise using the CFIR also provided feedback on development of the interview guides.

**Codebook Development.** An investigator from the coordinating center (LD) prepared an initial draft of the qualitative codebook based on the CFIR template and provided oversight of the pilot testing of the coding schema. Investigators from the ISWG reviewed and discussed the codes (domains and subconstructs) and definitions and iteratively refined the codebook. The codebook included a mapping of constructs from the original CFIR to CFIR 2.0 and notes whether a construct was removed, renamed, or relocated in CFIR 2.0 with instructions to refer to Damschroder et al. (2022) for definitions of new constructs.

**Data Collection.** Each RC conducted interviews and focus groups with members of their participating care teams and others involved in implementation (e.g., administrators, information technology staff). Interviews and focus groups were conducted in person or virtually. The timing of data collection varied with all RCs collecting data after implementation was complete, and two RCs (E2C2 and NU IMPACT) also collecting data during implementation. Each RC study protocol was approved by their affiliated Institutional Review Board (IRB).

RCs were asked to exchange one or two de-identified transcripts for the purposes of developing a shared method for qualitative coding and analysis that could be used across the consortium. The RCs and coordinating center first executed Data Transfer Agreements and obtained approval from their respective IRB to allow consortium investigators to exchange de-identified transcripts. Once the interview or focus group had been conducted and transcribed, the RCs labeled each transcript file with a unique identifier, data source (interview or focus group), and date of data collection, and uploaded the file to a secure data management repository. Prior to transfer, transcripts were de-identified by changing references to identifying information (e.g., names of individuals, organizations) to more generic terms.

**Coding and Data Integration.** The coding team for this work (JLR, JMG, AR, and AJC) included ISWG investigators from the three RCs who have expertise in qualitative research and significant experience using CFIR to code qualitative data obtained from pragmatic trials but had little familiarity with the intervention or with the contextual aspects of each other’s RC and care delivery settings when transcript coding. Each coder used the codebook to independently perform deductive coding in Microsoft Word by reading through the data and assigning codes to excerpts in each transcript. If applicable, investigators could assign more than one code, and excerpts assigned more than one code were a focus of subsequent discussion to achieve consensus and strengthen the codebook materials. Investigators worked together to code transcripts from each other’s RC (not only their RC). The process of combining the coded transcripts to evaluate agreement among coders was centralized with the coordinating center. Specifically, an investigator from the coordinating center (LD) merged coding from each transcript into a master file using NVivo 12 (QSR International) and generated reports in Microsoft Word to examine line-by-line disagreement in coding. Investigators from the RCs and coordinating center met monthly via Zoom between June 2021 to August 2023 to review discrepancies among coders and reach agreement across all RCs on how to apply CFIR codes (domain- and construct-level) for all 5 transcripts, making edits to the codebook as necessary to reflect coding decisions. Discussion was an important part of our process. Having an “outsider” perspective when coding forced investigators to consider the way in which position and subjectivity (i.e., reflexivity) played a role in their interpretation of the data. This critical reflection may have led “insider” investigators to think differently about their RC’s data. Representation of at least one investigator from each RC and the coordinating center was required for each meeting to occur. All meetings were recorded, and coding calibration and resolution of discrepancies was an iterative process involving multiple rounds of coding, team discussion and codebook revision with each transcript (Figure). One investigator from the coordinating center (LD) used the “Share Screen” function in Zoom to review the reports and make notes about coding decisions during the meetings.
