# Supplementary material for: Development of a method for qualitative data integration to advance implementation science within research consortia
Source: Implement Sci Commun. 2025 Feb 25;6:21. doi: 10.1186/s43058-025-00701-4 (PMC11853699; doi:10.1186/s43058-025-00701-4)
Supplement: Supplementary file 2 — Supplementary Material 2. [file 43058_2025_701_MOESM2_ESM.docx]

**Additional File 2**

**IMPACT Consortium Qualitative Coding Manual**

**Directions:**

- This codebook provides code definitions, inclusion and exclusion criteria for constructs from the CFIR.
- When selecting text excerpts to code, do code enough text so that the coded text would be able to stand on its own and make sense (i.e., code the question that precedes the interviewee’s response, code in complete sentences, or whole paragraphs if applicable).
- In general it is best to err on the side of coding all text (with the exception of informal/social conversations or topics that are clearly not relevant to the focus of the interview or focus group).
- Changes to constructs to harmonize with CFIR 2.0 have been noted where needed (e.g., if the construct was removed, renamed, or relocated). Analysts should refer to the CFIR 1.0 to 2.0 construct mapping document for complete definitions (Damschroder LJ, et al. The updated Consolidated Framework for Implementation Research based on user feedback. Implement Sci. 2022 Oct 29;17(1):75.).

**Definitions**

| 1. **Recommended Attributes/Case Classifications for Analysis** |  |
| --- | --- |
| 1. Research Center (RC) | Definition: Attribute Code (Research Center) used to group data for analysis (SIMPRO, E2C2, NU IMPACT) |
| 1. Clinical Site (SITE) | Definition: Attribute Code (Specific Site) used to group data for analysis. |
| 1. Intervention Role (ROLE) | Definition: Attribute Code (Physician, Nurse, Administrator, etc. …. ) used to group data for analysis. |
| 1. Cancer Specialty (SPECIALTY) | Definition: Attribute Code (GI, Lung, Gynecologic, Breast, etc.…. ) used to group data for analysis. |
| 1. Length of Exposure to Intervention (EXPOSURE) | Definition: Attribute Code (early, mid, late) used to group data for analysis. |

| 1. **Innovation Characteristics/**   **Intervention Characteristics**  **(CFIR 2.0)** | Use When: Interview participants are talking about the intervention and its components (see Additional File 2 for description of intervention components). Can be used to differentiate patient and clinician/staff perceptions of the intervention and its components.  Do Not Use: Interview participants talk about how the intervention was implemented/ strategies used to implement the intervention. Code under **Process** instead (see Additional File 2 for examples of implementation strategies).  Related Interview Questions:  NU IMPACT (note: these are “overview questions” that could generate response that could fall under any of the subcodes):   - Overall, do you think cPRO is effective in your setting? Why or why not? - How do you feel about cPRO continuing to be used in your setting? (Short-term? Long term?) |
| --- | --- |
| 1. Innovation   Source (CFIR 1.0 and 2.0) | Definition: Perception of key stakeholders about whether the innovation is externally or internally developed.  Inclusion Criteria: Include statements about the source of the innovation and the extent to which interviewees view the change as internal to the organization, e.g., an internally developed program, or external to the organization, e.g., a program coming from the outside. Note: May code and rate as “I” for internal or “E” for external.  Exclusion Criteria: Exclude or double code statements related to who participated in the decision process to implement the innovation to Engaging, as an indication of early (or late) engagement. Participation in decision-making is an effective engagement strategy to help people feel ownership of the innovation.  Related Interview Questions:  E2C2 (note: also included under Evidence Strength/Quality)   - What did you think of E2C2 when you first heard about it? - What are your impressions of the reason it was implemented? Was there a need for it? |
| 1. Evidence Strength & Quality (CFIR 1.0)/Innovation Evidence Base (CFIR 2.0) | Definition: Stakeholders’ perceptions of the quality and validity of evidence supporting the belief that the innovation will have desired outcomes.  Inclusion Criteria: Include statements regarding awareness of evidence and the strength and quality of evidence, as well as the absence of evidence or a desire for different types of evidence, such as pilot results instead of evidence from the literature.  Exclusion Criteria: Exclude or double code statements regarding the receipt of evidence as an engagement strategy to Engaging: Key Stakeholders.  Exclude or double code descriptions of use of results from local or regional pilots to Trialability.  Related Interview Questions:  SIMPRO   - What do your team members generally think about integrating ePROs into cancer care? - What did you think of eSyM when you first heard about it?   NU IMPACT   - What kind of information or evidence are you aware of that shows whether or not cPRO will work in your setting? E2C2 - What did you think of E2C2 when you first heard about it? [also Intervention Source] - What are your impressions of the reason it was implemented? Was there a need for it? [also intervention source] - How are you assessing the effectiveness of E2C2 in your clinic? Even if you are not currently assessing it, what would you want to know in order to judge whether it was effective? [also Reflecting & Evaluating] |
| 1. Relative Advantage (CFIR 1.0)/Innovation Relative Advantage (CFIR 2.0) | Definition: Stakeholders’ perception of the advantage of implementing the innovation versus an alternative solution.  Inclusion Criteria: Include statements that demonstrate the innovation is better (or worse) than existing ePRO programs or the status quo (usual care). For example, the intervention has addressed a particular problem for symptom reporting for patients.  Exclusion Criteria: Exclude statements that demonstrate a strong need for the innovation and/or that the current situation is untenable and code to Tension for Change.  Related Interview Questions:  SIMPRO   - What is your evaluation of the value of eSyM at your site so far? (also Reflecting & Evaluating)   NU IMPACT   - How does cPRO compare to other similar existing tools and programs in your setting? [Probe on: similarities, differences, perceived effectiveness...] - Is there another symptom management tool that people would rather implement?   E2C2   - How does E2C2 differ from the way you usually work with patients to manage symptoms? - Were there times when your team struggled seeing the value of E2C2? If so, how did you work through those issues? (note: may or may not generate relevant responses to this construct – e.g., relative advantage of E2C2 compared to usual care). |
| 1. Adaptability (CFIR 1.0)/Innovation Adaptability (CFIR 2.0) | Definition: The degree to which an innovation can be adapted, tailored, refined, or reinvented to meet local needs.  Inclusion Criteria: Include statements regarding the (in)ability to adapt the innovation to their context, e.g., complaints about the rigidity of the protocol. Suggestions for improvement can be captured in this code but should not be included in the rating process, unless it is clear that the participant feels the change is needed but that the program cannot be adapted. However, it may be possible to infer that a large number of suggestions for improvement demonstrates lack of compatibility, see exclusion criteria below.  Exclusion Criteria: Exclude or double code statements that the innovation did or did not need to be adapted to Compatibility.  Related Interview Questions:  SIMPRO   - Now that eSyM has gone live, what should be changed to make eSyM work better?   E2C2   - Thinking back to when you first started E2C2 compared to now, what kinds of changes have you made to E2C2 in order to make it better fit your clinic? - Have you needed to make adaptations in response to COVID-related conditions? - What kinds of future changes do you think are needed so that E2C2 works better for staff in your clinic? - Are there ways that you think E2C2 needs to adapt to better serve care teams that do symptom management? (Probe further on responses related to COVID) |
| 1. Trialability (CFIR 1.0)/Innovation Trialability (CFIR 2.0) | Definition: The ability to test the innovation on a small scale in the organization, and to be able to reverse course (undo implementation) if warranted.  Inclusion Criteria: Include statements related to whether the site piloted the innovation in the past or has plans to in the future, and comments about whether they believe it is (im)possible to conduct a pilot.  Exclusion Criteria: Exclude or double code descriptions of use of results from local or regional pilots to Evidence Strength & Quality.  Related Interview Questions:  No specific questions asked. |
| 1. Complexity (CFIR 1.0)/Innovation Complexity (CFIR 2.0) | Definition: Perceived difficulty of the innovation, reflected by duration, scope, radicalness, disruptiveness, centrality, and intricacy and number of steps required to implement.  Inclusion Criteria: Code statements regarding the complexity of the innovation itself.  Exclusion Criteria: Exclude statements regarding the complexity of implementation and code to the appropriate CFIR code, e.g., difficulties related to space are coded to Available Resources and difficulties related to engaging participants in a new program are coded to Engaging: Innovation Participants.  Related Interview Questions  E2C2   - Now that you have been using E2C2 (or at least you and your patients have had access to it) for [time], what are your impressions of it? - How does E2C2 make your work easier or harder? |
| 1. Design Quality & Packaging (CFIR 1.0)/Innovation Design (CFIR 2.0) | Definition: Perceived excellence in how the innovation is bundled, presented, and assembled.  Inclusion Criteria: Include statements regarding the quality of the materials and packaging or bundling of the intervention components.  Exclusion Criteria: Exclude statements regarding the presence or absence of materials and code to Available Resources.  Exclude statements regarding the receipt of materials as an engagement strategy and code to Engaging.  Related Interview Questions:  SIMPRO   - Have you reviewed the: Symptom questions? Tip Sheets? The training materials for patients? The training materials for staff? The engagement tracker? The population management dashboard? The symptom management “tip sheets”? RECORD each response as Y/N. If Y then ask:   - What is your view about the content of the (symptom questions, tip sheets, training materials for patients/staff)? What is your view about the design/deployment? Probe, how could the design be improved?   E2C2   - [No specific questions. Questions about impressions on components related to design could elicit feedback on design of education materials, provider dashboards, or other EHR tools] |
| 1. Cost (CFIR 1.0)/Innovation Cost (CFIR 2.0) | Definition: Costs of the innovation and costs associated with implementing the innovation including investment, supply, and opportunity costs.  Inclusion Criteria: Include statements related to the cost of the innovation and its implementation.  Exclusion Criteria: Exclude statements related to physical space and time, and code to Available Resources. In a research study, exclude statements related to costs of conducting the research components (e.g., funding for research staff, participant incentives).  Related Interview Questions:  No specific questions asked |
| 1. **Outer Setting** | Use When: Interview participants comment on the setting outside of the healthcare system that is implementing the ePRO intervention. |
| 1. Patient Needs & Resources of Those Served by the Organization (CFIR 1.0) /Roles Subdomain: Innovation Recipients/   Characteristics subdomain: need and culture (recipient-centeredness) (CFIR 2.0) | Definition: The extent to which patient needs of those served by the organization as well as barriers and facilitators to meet those needs, are accurately known and prioritized by the organization.  Inclusion Criteria: Include statements demonstrating (lack of) awareness of the needs and resources of patients. Analysts may be able to infer the level of awareness based on statements about: 1. Perceived need for the innovation based on the needs of patients and if the innovation will meet those needs; 2. Barriers and facilitators of patients to participating in the innovation; 3. Participant feedback on the innovation, i.e., satisfaction and success in a program. In addition, include statements that capture whether or not awareness of the needs and resources of patients influenced the implementation or adaptation of the innovation.  Exclusion Criteria: Exclude statements that demonstrate a strong need for the innovation and/or that the current situation is untenable and code to Tension for Change.  Exclude statements related to engagement strategies and outcomes, e.g., how innovation participants became engaged with the innovation, and code to Engaging: Innovation Participants.  Related Interview Questions:  SIMPRO   - What factors have made it easier for your patients to use eSyM? Has any factor made it more difficult? If so, what made it more difficult? - How has the COVID19 pandemic influenced implementation of eSyM at your site? [Note: Also could be anything in Inner Setting]   - Has it impacted the speed, scope, or timing of implementation? If so how?   - Has it impacted whether eSyM is viewed as a priority? If so how?   - Has it impacted leadership, staffing, or funding? If so how?   - Has it impacted physician or patient involvement? If so, how?   E2C2   - Are there other symptom management tools or resources that you discuss with patients? - How are patients at your site responding to E2C2? - What barriers might patients face in using E2C2?   COVID related questions:   - How has the COVID-19 environment impacted E2C2 implementation (e.g., PRO reporting)? [Note: also could be anything in Inner Setting] - Has COVID-19 impacted the way in which patients are communicating symptoms to their care teams? If so, how? - How has COVID-19 impacted the way in which patients manage their symptoms? - How has COVID-19 impacted symptoms for patients in your clinical area? Are there things that are unique to the patients you see in this clinical area (e.g., head and neck, lung, etc.) (or in this region/area)? - How has COVID-19 impacted the work that care teams do to support patients with symptoms? - Are there any other COVID-related impacts for patients managing cancer symptoms on their own? With the support of their care teams? |
| 1. Cosmopolitanism (CFIR 1.0)/Parternships & Connections (CFIR 2.0) | Definition: The degree to which an organization is networked with other external organizations.  Inclusion Criteria: Include descriptions of outside group memberships and networking done outside the organization.  Exclusion Criteria: Exclude statements about general networking, communication, and relationships in the organization, such as descriptions of meetings, email groups, or other methods of keeping people connected and informed, and statements related to team formation, quality, and functioning, and code to Networks & Communications.  Related Interview Questions:  No specific questions asked. |
| 1. Peer Pressure (CFIR 1.0)/External Pressure/Market Pressure (CFIR 2.0) | Definition: Mimetic or competitive pressure to implement an innovation, typically because most or other key peer or competing organizations have already implemented or are in a bid for a competitive edge.  Inclusion Criteria: Include statements about perceived pressure or motivation from other entities or organizations in the local geographic area or system to implement the innovation.  Exclusion Criteria:  Related Interview Questions  NU IMPACT   - To what extent did implementing the cPRO intervention provide an advantage for your organization compared to other organizations in your area? How could we maximize on this going forward? |
| 1. External Policy & Incentives (CFIR 1.0)/Policies & Laws (CFIR 2.0) | Definition: A broad construct that includes external strategies to spread innovations including policy and regulations (governmental or other central entity), external mandates, recommendations and guidelines, pay-for-performance, collaboratives, and public or benchmark reporting.  Inclusion Criteria: Include descriptions of external performance measures from the system. Include participant statements about COVID, telehealth, and insurance/reimbursement (these are things that are coming from outside of the institution and influencing the project).  Related Interview Questions:  SIMPRO   - Did any local, state, or national initiatives, policies, or guidelines influence eSyM implementation? If so, which ones?   E2C2   - Are there other factors—inside or outside of the organization your clinic/practice or even outside of Mayo Clinic—that make implementation of a system like this easier or harder? (Note: ACOs, survivorship plans, COPI certification, etc) |
| 1. Local Conditions (CFIR 2.0) | Definition: Economic, environmental, political, and/or technological conditions that enable the Outer Setting to support implementation and/or delivery of the innovation.  Related Interview Questions:  NU IMPACT   - Can you describe any other circumstances or issues outside of your practice/organization that impacted (or will impact) implementation of cPRO? |
| 1. **Inner Setting** | Use When: Interview participants comment on the setting within the implementing site, which includes the larger healthcare system that is implementing the ePRO intervention. |
| 1. Structural Characteristics (CFIR 1.0 and 2.0) | Definition: The social architecture, age, maturity, and size of an organization. Structural characteristics is very broad; need to differentiate between Policy, Physical Infrastructure, IT Infrastructure, etc.  Inclusion Criteria: Include statements pertaining to the Epic build, aspects related to staffing.  Related Interview Questions:  E2C2   - How has the infrastructure of your clinic (social or electronic architecture, IT support staff, EMR support staff, etc.) affected E2C2 implementation? - Have you needed to make any changes to infrastructure to incorporate E2C2? How easy or hard has it been to accommodate it? |
| 1. Networks & Communications (CFIR 1.0/Relational Connections (CFIR 2.0)/Communications (CFIR 2.0) | Definition: The nature and quality of webs of social networks, and the nature and quality of formal and informal communications within an organization.  Inclusion Criteria: Include statements about general networking, communication, and relationships in the organization, such as descriptions of meetings, email groups, or other methods of keeping people connected and informed, and statements related to team formation, quality, and functioning.  Exclusion Criteria: Exclude statements related to implementation leaders’ and users’ access to knowledge and information regarding using the program, i.e., training on the mechanics of the program and code to Access to Knowledge & Information.  Exclude statements related to engagement strategies and outcomes, e.g., how key stakeholders became engaged with the innovation and what their role is in implementation, and code to Engaging: Key Stakeholders.  Exclude descriptions of outside group memberships and networking done outside the organization and code to Cosmopolitanism.  Related Interview Questions:  No specific questions asked. |
| 1. Culture (CFIR 1.0 and 2.0) | Definition: Norms, values, and basic assumptions of a given organization.  Inclusion Criteria: Inclusion criteria, and potential sub-codes, will depend on the framework or definition used for “culture.”  Related Interview Questions:  No specific questions asked. |
| 1. Implementation Climate (CFIR 1.0) | Definition: The absorptive capacity for change, shared receptivity of involved individuals to an innovation, and the extent to which use of that innovation will be rewarded, supported, and expected within their organization.  Inclusion Criteria: Include statements regarding the general level of receptivity to implementing the innovation.  Exclusion Criteria: Exclude statements regarding the general level of receptivity that are captured in the sub-codes.  Related Interview Questions:  SIMPRO   - What factors have to be in place to increase the chances that eSyM implementation is a success?   **See below for additional questions**  NU IMPACT  **See below for questions**  E2C2   - Are there other factors—inside or outside of your clinic/practice or even outside of Mayo Clinic—that make implementation of a system like this easier or harder?   **See below for additional questions** |
| 1. Tension for Change (CFIR 1.0 and 2.0) | Definition: The degree to which stakeholders perceive the current situation as intolerable or needing change.  Inclusion Criteria: Include statements that (do not) demonstrate a strong need for the innovation and/or that the current situation is untenable, e.g., statements that the innovation is absolutely necessary or that the innovation is redundant with other programs. Note: If a participant states that the innovation is redundant with a preferred existing program, (double) code lack of Relative Advantage, see exclusion criteria below.  Exclusion Criteria: Exclude statements regarding specific needs of individuals that demonstrate a need for the innovation, but do not necessarily represent a strong need or an untenable status quo, and code to Needs and Resources of Those Served by the Organization.  Exclude statements that demonstrate the innovation is better (or worse) than existing programs and code to Relative Advantage.  Related Interview Questions:  No specific questions asked. |
| 1. Compatibility (CFIR 1.0 and 2.0) | Definition: The degree of tangible fit between meaning and values attached to the innovation by involved individuals, how those align with individuals’ own norms, values, and perceived risks and needs, and how the innovation fits with existing workflows and systems.  Inclusion Criteria: Include statements that demonstrate the level of compatibility the innovation has with organizational values and work processes, Include statements that the innovation did or did not need to be adapted as evidence of compatibility or lack of compatibility. For example, statements reflecting that an intervention is “compatible” would describe the intervention as better for the workflow vs. being a great intervention but individuals cannot determine how to implement.  Exclusion Criteria: Exclude or double code statements regarding the priority of the innovation based on compatibility with organizational values to Relative Priority, e.g., if an innovation is not prioritized because it is not compatible with organizational values.  Related Interview Questions:  SIMPRO   - Has eSyM impacted your workflow? How? - Does eSyM make your work easier or harder? Can you explan?   NU IMPACT   - How well does cPRO fit (or not) with existing work processes and practices in your setting? - How has cPRO been integrated into current processes?   E2C2   - Now that you have been using E2C2 (or at least you and your patients have had access to it) for [time], what are your impressions of it? - How does E2C2 make your work easier or harder? - How does E2C2 affect roles and responsibilities among clinical team members or other provider groups, if at all? |
| 1. Relative Priority (CFIR 1.0 and 2.0) | Definition: Individuals’ shared perception of the importance of the implementation within the organization.  Inclusion Criteria: Include statements that reflect the relative priority of the innovation, e.g., statements related to change fatigue in the organization due to implementation of many other programs. Include statements that discuss other priorities for patient care such as the need to reach out to new patients/all patients after starting a new treatment (not just those enrolled in the study).  Exclusion Criteria: Exclude or double code statements regarding the priority of the innovation based on compatibility with organizational values to Compatibility, e.g., if an innovation is not prioritized because it is not compatible with organizational values.  Related Interview Questions:  SIMPRO   - To what extent might the eSyM implementation take a backseat to other high-priority initiatives going on now?   NU IMPACT   - What other high-priority initiatives going on now? - How did cPRO fit in with the other initiatives? (Higher or lower priority?) - If other initiatives that use, are there any strategies or approaches that were effective and could be applied to cPRO?   E2C2   - What are your impressions of the reason it was implemented? Was there a need for it? (also innovation source and evidence strength and quality) |
| 1. Organizational Incentives & Rewards (CFIR 1.0)/Incentive Systems (CFIR 2.0) | Definition: Extrinsic incentives such as goal-sharing, awards, performance reviews, promotions, and raises in salary, and less tangible incentives such as increased stature or respect.  Inclusion Criteria: Include statements related to whether organizational incentive systems are in place to foster (or hinder) implementation, e.g., rewards or disincentives for staff engaging in the innovation.  Exclusion Criteria:  Related Interview Questions:  NU IMPACT   - Are there or can you think of any ways to incentivize use of cPRO? |
| 1. Goals & Feedback (CFIR 1.0)/Mission Alignment (CFIR 2.0) | Definition: The degree to which goals are clearly communicated, acted upon, and fed back to staff, and alignment of that feedback with goals.  Inclusion Criteria: Include statements related to the (lack of) alignment of implementation and innovation goals with larger organizational goals, as well as feedback to staff regarding those goals, e.g., regular audit and feedback showing any gaps between the current organizational status and the goal. Goals and Feedback include organizational processes and supporting structures independent of the implementation process. Evidence of the integration of evaluation components used as part of “Reflecting and Evaluating” into **on-going or sustained** organizational structures and processes may be (double) coded to Goals and Feedback.  Exclusion Criteria: Exclude statements that refer to the implementation team’s (lack of) assessment of the progress toward and impact of implementation, as well as the interpretation of outcomes related to implementation, and code to Reflecting & Evaluating. Reflecting and Evaluating is part of the implementation process; it likely ends when implementation activities end. It does not require goals be explicitly articulated; it can focus on descriptions of the current state with real-time judgment, though there may be an implied goal (e.g., we need to implement the innovation) when the implementation team discusses feedback in terms of adjustments needed to complete implementation.  Related Interview Questions:  SIMPRO   - To what extent is My Chart/patient portal enrollment an institutional priority? - To what extent is eSyM specifically an institutional priority? - Is eSyM implementation aligned with your institution’s goals? If yes, how? If not, why not? |
| 1. Learning Climate (CFIR 1.0)/Culture: Learning-Centeredness (CFIR 2.0) | Definition: A climate in which: 1. Leaders express their own fallibility and need for team members’ assistance and input; 2. Team members feel that they are essential, valued, and knowledgeable partners in the change process; 3. Individuals feel psychologically safe to try new methods; and 4. There is sufficient time and space for reflective thinking and evaluation.  Inclusion Criteria: Include statements that support (or refute) the degree to which key components of an organization exhibit a “learning climate.”  Related Interview Questions:  No specific questions asked. |
| 1. Readiness for Implementation (CFIR 1.0) | Definition: Tangible and immediate indicators of organizational commitment to its decision to implement an innovation.  Inclusion Criteria: Include statements regarding the general level of readiness for implementation.  Exclusion Criteria: Exclude statements regarding the general level of readiness for implementation that are captured in the sub-codes.  Related Interview Questions:  **See below for specific questions related to readiness for implementation** |
| 1. Leadership Engagement (CFIR 1.0) /Roles subdomain: high level and mid-level leaders/characteristics subdomain: motivation (CFIR 2.0) | Definition: Commitment, involvement, and accountability of leaders and managers with the implementation of the innovation.  Inclusion Criteria: Include statements regarding the level of engagement of organizational leadership.  Exclusion Criteria: Exclude or double code statements regarding leadership engagement to Engaging: Formally Appointed Internal Implementation Leaders or Champions *if* an organizational leader is also an implementation leader, e.g., if a director of primary care takes the lead in implementing a new treatment guideline. Note that a key characteristic of this Implementation Leader/Champion is that s/he is also an Organizational Leader.  Related Interview Questions:  E2C2   - What are your impressions of organizational support for E2C2? - Are there people you would describe as champions for E2C2 in your clinical area? How would you describe their role in implementation? |
| 1. Available Resources (CFIR 1.0 and 2.0) | Definition: The level of resources dedicated for implementation and on-going operations including physical space and time.  Inclusion Criteria: Include statements related to the presence or absence of more general and available resources specific to the innovation (e.g., staffing/employees) that is being implemented within an institution.  Exclusion Criteria: Exclude statements related to training and education and code to Access to Knowledge & Information.  Exclude statements related to the actual intervention cost and code to Intervention Characteristics – Cost.  Exclude statements related to the quality of materials and code to Design Quality & Packaging.  In a research study, exclude statements related to resources needed for conducting the research components (e.g., time to complete research tasks, such as IRB applications, consenting patients).  Related Interview Questions:  SIMPRO   - Do you have sufficient resources to implement and administer eSyM? (Probe on what is missing, which resources are critical, which are secondary, etc.”)   NU IMPACT   - What are the resources that are necessary to implement and administer cPRO? - What is missing, which resources are critical, which are secondary, which are “need to have” vs “nice to have”?   E2C2   - Do you have sufficient resources to implement and administer E2C2? (Probe on what is missing, which resources are critical, which are secondary, etc.”).) |
| 1. Access to Knowledge & Information (CFIR 1.0 and 2.0) | Definition: Ease of access to digestible information and knowledge about the innovation and how to incorporate it into work tasks.  Inclusion Criteria: Include statements related to implementation leaders’ and users’ access to knowledge and information regarding use of the symptom management system, i.e., training on the mechanics of the intervention. Include statements that relate to one step (or wave) learning about the intervention from prior steps/waves in the stepped wedge trial. Include action oriented statements about how the intervention works in the inner setting (i.e., do they have information to share with patients).  Exclusion Criteria: Exclude statements related to engagement strategies and outcomes, e.g., how key stakeholders became engaged with the innovation and what their role is in implementation, and code to Engaging: Key Stakeholders.  Exclude statements about general networking, communication, and relationships in the organization, such as descriptions of meetings, email groups, or other methods of keeping people connected and informed, and statements related to team formation, quality, and functioning, and code to Networks & Communications.  Exclude broad statements about the intervention (i.e., do they think it is beneficial or not). Code to Individual Characteristics: Knowledge & Beliefs.  Related Interview Questions:  SIMPRO   - Do you have a way to get the information you need about eSyM? (Probe on what is missing, which resources are critical, which are secondary, etc.)   NU IMPACT   - What are the materials needed to train and provide continued support for those involved in cPRO implementation? What are greatest needs/additional needs?   E2C2   - Are there components that you didn’t know about or need more information about? - How do you feel about the training and resources you received in regards to feeling prepared to implement the changes? |
| 1. **Individuals**   **(CFIR 1.0 and 2.0)** | Use When: Participants talks about the roles and characteristics of individuals.  Related Interview Questions:  SIMPRO   - How familiar are you with eSyM? - How has the COVID19 pandemic influenced attitudes towards eSyM at your site?   E2C2   - What do you think are the most critical components of E2C2? Why? - Which components do you think will be most effective for helping patients with symptoms? - Do patients need new types of symptom support during COVID? What is still missing? - What tools or resources are needed by the care teams that are supporting patients during these times? - What components are you using the least or find to be of the least value? What could be done to make those more useful?   NU IMPACT   - In your opinion, do the people in your clinics have the appropriate skills, availability, and motivation to continue use of cPRO? |
| 1. Knowledge & Beliefs about the Innovation (CFIR 1.0) | Definition: Individuals’ attitudes toward and value placed on the innovation, as well as familiarity with facts, truths, and principles related to the innovation.  Inclusion Criteria: Include statements when participant is discussing general feelings about the intervention (e.g., “I was excited about it rolling out”).  Exclusion Criteria: Exclude statements related to familiarity with evidence about the innovation and code to Evidence Strength & Quality. |
| 1. Self-efficacy (CFIR 1.0) | Definition: Individual belief in their own capabilities to execute courses of action to achieve implementation goals. |
| 1. Individual Stage of Change (CFIR 1.0) | Definition: Characterization of the phase an individual is in, as s/he progresses toward skilled, enthusiastic, and sustained use of the innovation.  Inclusion Criteria: Include statements that reflect participants excitement and enthusiasm at the start of implementation and during implementation. For example, “I was excited about rolling it out” could be coded to both Knowledge & Beliefs and Stage of Change. |
| 1. Individual Identification with Organization (CFIR 1.0) | Definition: A broad construct related to how individuals perceive the organization, and their relationship and degree of commitment with that organization. |
| 1. Other Personal Attributes (CFIR 1.0) | Definition: A broad construct to include other personal traits such as tolerance of ambiguity, intellectual ability, motivation, values, competence, capacity, and learning style. |
| 1. **Process**   **(CFIR 1.0 and 2.0)** | Use when: Interview participants talk about how the intervention was implemented/ strategies used to implement the intervention (see Additional File 2 for examples of implementation strategies).  Do not use: Interview participants are talking about the intervention and its components (see Additional File 2 for description of intervention components). Code as Intervention Characteristics.  Related Interview Questions:  NU IMPACT   - What are your thoughts on how cPRO has been implemented to date?   Probe: How well do you think it was implemented? |
| 1. Planning (CFIR 1.0 and 2.0) | Definition: The degree to which a scheme or method of behavior and tasks for implementing an innovation are developed in advance, and the quality of those schemes or methods.  Inclusion Criteria: Include evidence of pre-implementation diagnostic assessments and planning, as well as refinements to the plan.  Related Interview Questions:  E2C2   - No questions specifically but some questions when used with key stakeholders (instead of care team members) may elicit responses to this domain. |
| 1. Engaging (CFIR 1.0 and 2.0) | Definition: Attracting and involving appropriate individuals in the implementation and use of the innovation through a combined strategy of social marketing, education, role modeling, training, and other similar activities.  Inclusion Criteria: Include statements related to engagement strategies and outcomes, i.e., if and how staff and innovation participants became engaged with the innovation and what their role is in implementation. Note: Although both strategies and outcomes are coded here, the outcome of engagement efforts determines the rating, i.e., if there are repeated attempts to engage staff that are unsuccessful, or if a role is vacant, the construct receives a negative rating. In addition, you may also want to code the "quality" of staff - their capabilities, motivation, and skills, i.e., how good they are at their job, and this data affects the rating as well.  Exclusion Criteria: Exclude statements related to specific sub constructs, e.g., Champions or Opinion Leaders.  Exclude or double code statements related to who participated in the decision process to implement the innovation to Innovation Source, as an indicator of internal or external innovation source.  Related Interview Questions:  SIMPRO   - What else could be done to engage patients? - What else could be done to engage staff?   NU IMPACT  **See below for questions related to engaging**  E2C2   - How would you describe buy-in for E2C2 among your team? - What strategies have you used to increase adoption and use among clinical team members (e.g., training for staff, reminders, changes to processes)? What strategies have you used to increase adoption and use among patients? |
| 1. Opinion Leaders (CFIR 1.0)/Roles Subdomain: Opinion Leaders (CFIR 2.0) | Definition: Individuals in an organization that have formal or informal influence on the attitudes and beliefs of their colleagues with respect to implementing the innovation.  Inclusion Criteria: Include statements related to engagement strategies and outcomes, e.g., how the opinion leader became engaged with the innovation and what their role is in implementation. Note: Although both strategies and outcomes are coded here, the outcome of efforts to engage staff determines the rating, i.e., if there are repeated attempts to engage an opinion leader that are unsuccessful, or if the opinion leader leaves the organization and this role is vacant, the construct receives a negative rating. In addition, you may also want to code the "quality" of the opinion leader here - their capabilities, motivation, and skills, i.e., how good they are at their job, and this data affects the rating as well.  Related Interview Questions:  SIMPRO   - Who are the key leaders who need to be on board to make eSyM implementation succeed at your institution? - To what extent are they on board?   NU IMPACT   - Who are the people in your clinic/region that were most involved in the implementation of cPRO? - Who are the right people to be involved going forward? |
| 1. Formally Appointed Internal Implementation Leaders (CFIR 1.0)/Roles Subdomain: Implementation Leads (CFIR 2.0) | Definition: Individuals from within the organization who have been formally appointed with responsibility for implementing an innovation as coordinator, project manager, team leader, or other similar role.  Inclusion Criteria: Include statements related to engagement strategies and outcomes, e.g., how the formally appointed internal implementation leader became engaged with the innovation and what their role is in implementation. Note: Although both strategies and outcomes are coded here, the outcome of efforts to engage staff determines the rating, i.e., if there are repeated attempts to engage an implementation leader that are unsuccessful, or if the implementation leader leaves the organization and this role is vacant, the construct receives a negative rating. In addition, you may also want to code the "quality" of the implementation leader here - their capabilities, motivation, and skills, i.e., how good they are at their job, and this data affects the rating as well.  Exclusion Criteria: Exclude or double code statements regarding leadership engagement to Leadership Engagement *if* an implementation leader is also an organizational leader, e.g., if a director of primary care takes the lead in implementing a new treatment guideline.  Related Interview Questions:  E2C2   - No questions specifically but some questions when used with key stakeholders (instead of care team members) may elicit responses to this domain. |
| 1. Champions (CFIR 1.0)/Roles Subdomain: Implementation Leads (CFIR 2.0) | Definition: “Individuals who dedicate themselves to supporting, marketing, and ‘driving through’ an [implementation]”, overcoming indifference or resistance that the innovation may provoke in an organization.  Inclusion Criteria: Include statements related to engagement strategies and outcomes, e.g., how the champion became engaged with the innovation and what their role is in implementation. Note: Although both strategies and outcomes are coded here, the outcome of efforts to engage staff determines the rating, i.e., if there are repeated attempts to engage a champion that are unsuccessful, or if the champion leaves the organization and this role is vacant, the construct receives a negative rating. In addition, you may also want to code the "quality" of the champion here - their capabilities, motivation, and skills, i.e., how good they are at their job, and this data affects the rating as well.  Exclusion Criteria: Exclude or double code statements regarding leadership engagement to Leadership Engagement *if* a champion is also an organizational leader, e.g., if a director of primary care takes the lead in implementing a new treatment guideline.  Related Interview Questions:  NU IMPACT   - Who are the people in your organization who are essential/important champions for the continued implementation of cPRO?   E2C2   - Are there people you would describe as champions for E2C2 in your clinical area? How would you describe their role in implementation? |
| 1. External Change Agents (CFIR 1.0)/ Roles Subdomain: Implementation Facilitators (CFIR 2.0) | Definition: Individuals who are affiliated with an outside entity who formally influence or facilitate innovation decisions in a desirable direction.  Inclusion Criteria: Include statements related to engagement strategies and outcomes, e.g., how the external change agent (entities outside the organization that facilitate change) became engaged with the innovation and what their role is in implementation, e.g., how they supported implementation efforts. Note: Although both strategies and outcomes are coded here, the outcome of efforts to engage staff determines the rating, i.e., if there are repeated attempts to engage an external change agent that are unsuccessful, or if the external change agent leaves their organization and this role is vacant, the construct receives a negative rating. In addition, you may also want to code the “quality” of the external change agent here – their capabilities, motivation, and skills, i.e., how good they are at their job, and this data affects the rating as well.  Exclusion Criteria: Note: It is important to clearly define what roles are external and internal to the organization. Exclude statements regarding facilitating activities, such as training in the mechanics of the program, and code to Access to Knowledge & Information *if* the change agent is considered internal to the study, e.g., a staff member at the national office. If the study considers this staff member internal to the organization, it should be coded to Access to Knowledge & Information, even though their support may overlap with what would be expected from an External Change Agent.  Related Interview Questions:  No specific questions asked. |
| 1. Executing (CFIR 1.0)/Doing (CFIR 2.0) | Definition: Carrying out or accomplishing the implementation according to plan.  Inclusion Criteria: Include statements about the implementation process. Include statements that demonstrate how implementation occurred with respect to the implementation plan. Note: Executing is coded very infrequently due to a lack of planning. However, some studies have used fidelity measures to assess executing, as an indication of the degree to which implementation was accomplished according to plan.  Related Interview Questions:  SIMPRO   - How do you think the eSyM implementation process has worked so far?   - What has worked well/been effective?   - What hasn’t worked well or could be improved?   NU IMPACT   - What are the strategies/approaches/tools used that will most help you to continue to implement cPRO? |
| 1. Reflecting & Evaluating (CFIR 1.0 and 2.0) | Definition: The degree to which goals are clearly communicated, acted upon, and fed back to staff, and alignment of that feedback with goals.  Inclusion Criteria: Include statements related to the (lack of) alignment of implementation and innovation goals with larger organizational goals, as well as feedback to staff regarding those goals, e.g., regular audit and feedback showing any gaps between the current organizational status and the goal. Goals and Feedback include organizational processes and supporting structures independent of the implementation process. Evidence of the integration of evaluation components used as part of “Reflecting and Evaluating” into **on-going or sustained** organizational structures and processes may be (double) coded to Goals and Feedback.  Exclusion Criteria: Exclude statements that refer to the implementation team’s (lack of) assessment of the progress toward and impact of implementation, as well as the interpretation of outcomes related to implementation, and code to Reflecting & Evaluating. Reflecting and Evaluating is part of the implementation process; it likely ends when implementation activities end. It does not require goals be explicitly articulated; it can focus on descriptions of the current state with real-time judgment, though there may be an implied goal (e.g., we need to implement the innovation) when the implementation team discusses feedback in terms of adjustments needed to complete implementation.  Related Interview Questions:  SIMPRO   - What is your evaluation the value of eSyM at your site so far? - What does a site that is considering adopting eSyM need to know?   NU IMPACT   - What goals or metrics do you think are most appropriate for your organization/unit? (e.g., completion rates, referrals)   E2C2   - How are you assessing the effectiveness of E2C2 in your clinic? Even if you are not currently assessing it, what would you want to know in order to judge whether it was effective? |

| **Defining Intervention Components vs. Implementation Strategies** | | | | | |
| --- | --- | --- | --- | --- | --- |
| **NU IMPACT** | | **E2C2** | | **SIMPRO** | |
| **Clinical Intervention** | **Implementation Strategies** | **Clinical Intervention** | **Implementation Strategies** | **Intervention** | **Implementation Strategies** |
| - EHR integrated cancer PRO symptom monitoring program (cPRO) - Targeted web-based tool named “My NM Care Corner” – includes self management materials - Tabular and graphic feedback on reported symptoms in the EHR patient portal (w/in MyChart) | - Clinical alerts (to providers) via EHR in-basket messages for severe symptoms - Automated reinforcement emails, calls, text messages to access My NM Care Corner (to patients) - Nurse outreach to patients for cPRO completion - Working with regions and Quality to identify OL and PCs and develop educational materials for the trainings; Kick-off meetings; development of guide/slides - Development of poster, pamphlets/flyers, and web text for patients - Develop video, slide deck, 1 page reference doc, training materials, and quarterly newsletter for clinicians, other clinic staff, and administrators - cPRO scores are graphically displayed in Epic for clinicians to view | - **Symptom Burden and Patient Preferences Assessment**: Automated requests are sent to patients to complete PRO assessments; based on level of symptom burden on assessments, patients receive the next steps in care (2) or (3) - **Patient Self-Management Resources:** For patients experiencing moderate symptoms in (1), clinical sites distribute educational materials to patients through postal mail or in person to support symptom self-management. EPIC also includes a patient dashboard in the patient portal for patients to track their personal symptom burden. - **Nurse-Led Facilitation of Guideline Concordant Team-Based Care:** For patients experiencing severe symptoms in (1), they are asked if they would like to talk with a Nurse Symptom Care Manager (RN-SCM). RN-SCMs support collaborative care as follows: (a) educate patients on evidence-based management strategies; (b) facilitate communication between patients and care team; (c) refer patients to specialists; (d) prescribe algorithm-based patient treatments and/or facilitate medication prescribing/ management with providers. | - **Offer multimodal PRO collection:** PROs collected via patient portal, tablets, interactive voice response. - **Practice Facilitation:** Research team: (a) identifies and trains clinical champions in use of E2C2 tools ; (b) familiarizes the clinical teams with RN SCMs; (c) identifies and trains care team members to be practice facilitators. - **Point of Care Prompts**: Best Practice Alerts triggered in EPIC for clinicians for patients with severe symptoms. - **Audit and feedback :** Research team develops and distributes monthly newsletters that include symptom management data. EPIC includes a clinical dashboard for clinicians to track patients’ symptom burden. - **Develop and Distribute Education Materials for Care Teams:** The research team developed and distributed the following : (a) clinician cheat sheet with key contacts, numbers, and Epic instructions for symptom management tools; (b) reminder cards are attached by roomers to rooming sheets to remind clinicians to describe symptom management resources/processes to patients. | - EHR integrated cancer PRO symptom monitoring program (eSYM) - Prompting patients to report symptoms at pre-defined intervals; - Directing patients to self-care educational tip sheet library in response to reported symptoms - Providing normative feedback and anticipatory guidance to patients about symptom profiles; - Clinicians calling patients to address reported severe symptoms according to institutional guidelines for symptom management | - Clinician training materials developed and circulated - Frequent engagement calls with clinical departments to review KPIs, workflows, and strategies - Assisting nurses with eSyM results review - Coached nurses on what to look for in eSyM dashboards - Hire new staff to assist program - Leverage the Research Coordinator to review reports and provide feedback to the department. - Administrative staff conduct email/phone check-ins with pts to assist with eSyM setup - Emailed reminders and mailed program flyers to reengage staff - Tracking/presenting symptom profiles over time in an EHR-embedded flowsheet; - Triggering delivery of self-care coaching in response to mild to moderate symptoms; - Integrating secure smartphone or tablet apps for patient data reporting. - Alerting patients to contact their clinicians in response to severe symptom reports - Alerting clinicians about patients with severe symptoms - Facilitating symptom burden monitoring of user-defined patient cohorts via dashboards in the EHR |
