# Supplementary material for: Development of a method for qualitative data integration to advance implementation science within research consortia
Source: Implement Sci Commun. 2025 Feb 25;6:21. doi: 10.1186/s43058-025-00701-4 (PMC11853699; doi:10.1186/s43058-025-00701-4)
Supplement: Supplementary file 3 — Supplementary Material 3. [file 43058_2025_701_MOESM3_ESM.docx]

| **Additional File 3.**  **Coding Issues**  **Definitions and Inclusion Criteria for Roles Subdomain** | | | | |
| --- | --- | --- | --- | --- |
| **Construct** | **Definition** | **Inclusion Criteria** | | |
|  |  | **E2C2** | **SIMPRO** | **NU IMPACT** |
| A. High-level Leaders | Individuals with a high level of authority, including key decision-makers, executive leaders, or directors. | Cancer Center and system level leadership | PIs, Institution medical directors, Hospital leadership | PIs, university and hospital leadership |
| B. Mid-level Leaders | Individuals with a moderate level of authority, including leaders supervised by a high-level leader and who supervise others. | Nursing leadership and management | eSyM Champions, Co-Investigators, Project managers, nursing directors | Practice managers, in-clinic leaders |
| C. Opinion Leaders | Individuals with informal influence on the attitudes and behaviors of others. | Division and care team chairs and clinical leads | Nursing staff, physicians, APPs, hospital administration | Nursing staff, physicians, APPs, MAs, social workers, dieticians – depending on context |
| D. Implementation Facilitators | Individuals with subject matter expertise who assist, coach, or support implementation. | Symptom Sages for each tumor group, Epic informatics support for clinical teams | PIs, Co-Investigators, Medical directors, Nurse directors, project managers | Research team members, clinic admin staff |
| E. Implementation Leads | Individuals who lead efforts to implement the innovation. | E2C2 PI and Co-I | PIs, Project managers | PIs, operational leads (practice managers, quality directors, director of living well, program managers; depends on the region), Physician Champions |
| F. Implementation Team Members | Individuals who collaborate with and support the Implementation Leads to implement the innovation, ideally including Innovation Deliverers and Recipients. | Members of the clinical team (including providers), support for implementation facilitators, members of the implementation workgroup | Research staff, eSyM champions (select physicians), nurses (including Oncology Nurse Navigators) site technical staff (informatics/Epic)* | Physicians (non-champions), Social Workers, nurses/MAs, Patient Service Rep/Patient Advocate Service, Quality Leaders, community health workers |
| G. Other Implementation Support | Individuals who support the Implementation Leads and/or Implementation Team Members to implement the innovation. | Data, Epic, and study coordination teams (e.g., report development, scheduling, education materials) | Nursing directors, physicians, Co-Investigators, eSyM Champions, | Information Services team members, analytics team members |
| H. Innovation Deliverers | Individuals who are directly or indirectly delivering the innovation. | Symptom Care Managers (SCMs), as well as health coaches, physical therapists, and social workers (when delivering the intervention) | PIs, project managers, research staff, Epic technical teams | Social workers, dieticians, nurses/MAs/providers who are alerted |
| I. Innovation Recipients | Individuals who are directly or indirectly receiving the innovation. | Patients | Patients (and proxies), nursing staff, physicians | Patients |

*NOTE: Most physicians at the SIMPRO institutions were not implementation team members, but instead were predominantly serving as clinicians and provided some occasional implementation support; they have been classified here as ‘other implementation support’. SIMPRO also had explicitly identified eSyM champions (some select physicians) who were designated as implementation team members. Oncology Nurse Navigators job role falls under the broad ‘nurse’ category. There are many different nursing roles at SIMPRO sites (e.g., infusion nurses, surgical nurses, oncology nurse navigators, triage nurses).

| **Additional Examples of Coding Issues by CFIR Domain and Construct** | | | | |
| --- | --- | --- | --- | --- |
| **Transcript Source** | **Examples of Coding Issues** | **Quote** | **Coding Discrepancy Resolution** | **CFIR Domain/Construct** |
| SIMPRO Interview | Clarify whether references to messaging prompts to patients to report symptoms are part of the intervention or implementation strategy. | “Interviewer: I know the eSyM^1^ assignment goes out the day after day one, cycle one. But the current messaging doesn’t remind patients to do it until day four, but they could do it on day two, without actually changing anything.  Respondent: Yes. So, I think there’s definitely room for us to improve that messaging and maybe shift it up a little bit, so that you could align those calls.” | Prompting patients to report symptoms at pre-defined intervals is a part of the intervention. | Innovation/Adaptability |
| NU IMPACT Focus Group | Lack of clarity about when to apply the “*External Pressure*” codes. | “We do, for the Commission on Cancer, have to screen for a couple of things, but what are going to be the things with patient reported outcomes that clinicians feel like they need? And I don't think that they're mutually the same.” | Code statements about the influence of cancer guidelines, accreditation, and cancer program models on PRO reporting under “External Pressure” and “Performance-Measurement Pressure” | Outer Setting/External Pressure/Performance Measurement Pressure |
| SIMPRO Interview  E2C2 Interview | Lack of clarity about when to apply “*Relative Priority*” and “*Compatibility*” codes. | “So, I think for us, it’s tough, because there’s also the priority of new patient calls. And calling after first chemos, and things like that. That I think trying to figure out what is the actual priority, and what is gonna give us the most benefit for patient care.”  “I haven’t seen it make it any harder. That’s for sure. Also, the symptom care managers have teed up orders for us, which is a blessing. That’s something else that has made my life easier, whether it be referrals to palliative care or certain medication, anti-emetics, or whatever the case may be, and I've always reviewed them but felt that they were very appropriate, and so that has saved me some time.” | Code statements about other priorities for patient care under “relative priority”.  Code statements reflecting the intervention as better for clinical workflow (as opposed to it being a great intervention but individuals cannot determine how to implement it) under “compatibility”. | Inner Setting/Relative Priority  Inner Setting/Compatibility |
| NU IMPACT Focus Group | Lack of clarity about which “*Roles*” subdomain code and corresponding “*Characteristics”* subdomain code to use for physicians | “If I just got better completion of it [cPRO]^2^ with in person, in clinic. I don't need three days in advance because the ones that are alerting me, I know that they're severe. I know it. They're my sick patients. It's not that interesting. The severe pain ones, I already know they're in severe pain.” | Code statements where physicians discuss aspects of patient care that the intervention does (does not) address) under “implementation team member” and “need” | Individual/Role/  Implementation Team Member/Need |
| E2C2 Interview | Determine whether references to rack cards/reminder cards or action planning tools are part of the intervention or an implementation strategy. | “I have not, personally, used the action planning tool. But the one prior to that – the one you showed – yeah, that one, the rack card because, again, it kind of lists the websites and phone numbers and so I have used that more often than the other.” | Developing and distributing educational materials for the Care Teams are part of the implementation strategy. | Process/Engaging/  Innovation Deliverers |

^1^eSyM is the electronic symptom monitoring intervention implemented by SIMPRO.

^2^cPRO is the electronic symptom monitoring intervention implemented by NU IMPACT.
